# Assessing Medication Adherence Barriers to Short-Term Oral Antibiotic Treatment in Primary Care—Development and Validation of a Self-Report Questionnaire (BIOTICA)

**DOI:** 10.3390/ijerph18157768

**Published:** 2021-07-22

**Authors:** Melanie Haag, Kurt E. Hersberger, Isabelle Arnet

**Affiliations:** Pharmaceutical Care Research Group, Department of Pharmaceutical Sciences, University of Basel, 4001 Basel, Switzerland; kurt.hersberger@unibas.ch (K.E.H.); isabelle.arnet@unibas.ch (I.A.)

**Keywords:** medication adherence, medicines optimization, patient-centred pharmaceutical care, pharmaceutical care, responsibilities/roles in pharmaceutical care

## Abstract

Background: Pharmacists are increasingly involved in strategies to fight antimicrobial resistance by ensuring optimised antibiotic (AB) use, including adherence support. Successful adherence interventions should be tailored to patients’ barriers and validated instruments are needed. This study aimed to identify adherence barriers to AB treatment, develop a self-report questionnaire, and validate it in outpatients. Method: Adherence barriers were identified through a systematic literature search and focus group discussion. Unmodifiable and irrelevant barriers were excluded from further processing. A validation study assessed the questionnaire’s internal reliability and construct validity by comparing the questionnaire’s score with electronically monitored adherence data. Results: A 15-item questionnaire was created. Overall, 68 patients were included in the construct validation analysis (60.3% female). The mean consecutive taking adherence was 88% and the most frequently reported barriers were “worries about side-effects” (37%) and “having swallowing difficulties” (19%). Three items were excluded from the questionnaire, which was supported by an increase of Cronbach’s alpha (0.69 to 0.70). The 12-item version’s score correlated significantly with medication adherence rate (r = −0.34, *p* < 0.01). Conclusion: The self-report questionnaire is a reliable and valid tool to pre-emptively assess adherence barriers in outpatients prescribed ABs. In the future, appropriate adherence interventions can be matched to barriers and tested in a pilot intervention study.

## 1. Introduction

Medication adherence is a behaviour defined as the “process by which patients take their medications as prescribed” [1]. The process is divided into three phases, comprising initiation (that is, “when the patient takes the first dose of a prescribed medication”), discontinuation (that is, “when the patient stops taking the prescribed medication, for whatever reasons”) and implementation (that is, the “extent to which a patient’s actual dosing corresponds to the prescribed dosing regimen”) [1]. From longitudinal studies in patients suffering from a chronic disease such as hypertension, we know that approximately 50% of them stop taking their antihypertensive medicine within one year [2]. However, less is known about medication adherence during acute illnesses such as infectious diseases requiring short-term antibiotic (AB) therapies. Studies conducted in this field report a wide range of medication adherence rates from as low as 28% up to 100% [3,4]. According to an international survey with oral AB treatment, non-adherence occurred in all three phases with 3.7% non-initiation, 6.1% poor implementation, and 11% discontinuation [5]. Two studies targeted individuals visiting a community pharmacy with a prescription for oral ABs. The first study from Portugal reports a taking adherence of 55% that was assessed with a self-reported 8-items scale 30 days after treatment completion [6]. The second study was conducted in fourteen Maltese community pharmacies and reported a ta-king adherence of 76% before and 90% after an educational intervention [7]. Patients were asked to report the number of pills left over, and any leftovers would justify being non-adherent.

Non-adherence is a complex and multidimensional phenomenon related to healthcare system, social/economic, therapy-related, patient-related, and condition-related factors [8]. Those factors are further referred to as adherence barriers. Barriers to oral AB therapies include, among others, “feeling better”, “too busy in study or work“, side-effects”, “forgetfulness”, and “save some doses for the future” [5,7,9]. In the past, medication adherence enhancing interventions showed mixed results, mainly because medication adherence enhancing interventions were not tailored to patients’ needs [10,11,12,13]. Allemann et al. suggested that successful medication adherence intervention should target non-adherent patients and be tailored to individual adherence barriers (e.g., no social support or forgetfulness) [14,15,16].

Non-adherence to oral ABs can lead to poor clinical outcomes [17], increased healthcare costs due to enlarged healthcare consumption and hospital stays [9,10], and increased risk of emerging antimicrobial resistance due to sub-therapeutic medicine concentrations [18]. The American Centre for Disease Control lists “patients not taking antibiotics as prescribed”, a manifestation of non-adherence, as one of six causes for antibiotic resistance [19]. Antibiotic resistance is a growing public health problem, and pharmacists are increasingly involved in antimicrobial resistance (AMR) programmes by providing pharmaceutical care services [19,20,21]. The term pharmaceutical care was recently defined as “pharmacist’s contribution to the care of individuals in order to optimise medicines use and improve health outcomes” [22]. This underpins community pharmacists’ role as “contributing to improving effectiveness of health care systems and public health” and introduces a shift from a product-centred role to a provider of patient-centred services [23,24]. For example, pharmacists can advise about the risk of prescribing and withholding ABs for patients seeking advice for common infections [25]. Through their frequent interactions with patients filling prescriptions for oral ABs, pharmacists might be ideally positioned to address medication adherence while dispensing oral AB medicine. To take responsibility, pharmacists need valid and reliable instruments to assess medication adherence barriers that follow the SMART criteria that are specific, measurable, attainable, relevant, and time-bound.

Self-reported questionnaires are used regularly in medication adherence research because they are inexpensive and not time-consuming. Recently, questionnaires assessing adherence barriers have been developed, such as the “Medication Adherence Reasons Scale (MAR)” [26], the “Adherence Barriers Questionnaire (ABQ)” [27,28], and “Identify barriers to medication adherence questionnaire (IMAB-Q) [29]. However, none of these instruments fulfilled our need for specific barriers to oral AB intake, assessed before treatment initiation. Therefore, we decided to create a specific tool. This study aimed to identify medication adherence barriers to oral AB therapies, develop a self-report questionnaire, and validate it in an outpatient setting.

## 2. Materials and Methods

### 2.1. Literature Search

We conducted a systematic literature search according to PRISMA guidelines to identify medication adherence barriers in PubMed, EMBASE, and CINHAL up to 3 May 2019. The complete search strategy used for all three databases is described in Supplement 1. Eligibility criteria for study inclusion were outpatients > 18 years, intake of oral ABs, assessment of medication adherence, and reporting on medication adherence barriers. The assessment for eligibility was performed independently by two researchers (MH, AS). Discrepancies were solved by discussion and when no agreement could be reached, a third researcher (IA) decided. MH and AS extracted data regarding first author, year, country, study design, number of participants, age, indication, oral AB agent, medication adherence rate, and barriers of included articles. The two researchers classified medication adherence barriers into modifiable barriers (i.e., changeable through measures taken by someone) and unmodifiable barriers (i.e., that cannot be changed). Synonyms were removed and similar topics were conflated. Modifiable barriers were categorised according to the Theoretical Domains Framework (TDF) adapted by Allemann et al. [14].

### 2.2. Focus Group Discussion

Two researchers (MH, AS) conducted a focus group discussion (FGD) to identify medication adherence barriers to ABs most relevant for outpatients. Eligible participants were individuals > 18 years who had taken oral ABs for 3–30 days within the past six months. Recruitment was performed through 3 channels with distribution of a flyer containing detailed information on the FGD: During the public lecture “Microbes among us” at the University of Basel on 12 February 2019; to clients in five community pharmacies in Basel and Bern, and through the Pharmablog reaching all students of the Department of Pharmaceutical Sciences of Basel. Personal contacts were allowed. A financial incentive (CHF 50) was given as compensation. Medication adherence barriers perceived by parti-cipants were collected during a one-hour moderated discussion. Additional barriers retrieved from the literature search were presented to the participants who had to prioritise all barriers by rating them according to their relevance. For the rating, participants could distribute 33 points to relevant barriers in any combination (from zero to 33). Barriers ranking in the lowest quartile were excluded from further processing.

### 2.3. Content Validity

Content validity of the questionnaire was assessed according to Almanasreh et al. [30]. German-speaking experts in the field of medication adherence who were working in academia or a clinical setting were recruited from our research network and asked to participate in a content validity survey. The survey was created and disseminated using Google Forms. We asked experts to rate each item on a four-point Likert-scale according to its relevance, representativeness, clarity, ambiguity, and comprehensiveness. A free text option was available after each item to add comments. For each item, content validity index (I-CVI) and kappa coefficient were calculated. Items with an I-CVI < 0.78 and a kappa value < 0.4 were revised or excluded if irrelevant [30].

### 2.4. Construct Validity

To investigate the psychometric properties of the developed questionnaire, a cross-sectional, non-interventional study was performed with outpatients taking oral ABs. We selected community pharmacists and general practitioners (GP) in our network who were experienced in performing research studies through former work with our group [31]. They were located in cities and rural areas and are representative for German speaking part of Switzerland. Participating pharmacists and GPs recruited eligible patients during daily practice. Inclusion criteria of patients to participate in the study were ≥18 years, having a prescription for oral ABs for 3–30 days, self-managing medication, able to read and understand German, and agreeing to record every AB intake with an electronic device [32]. Exclusion criteria were pregnancy and people who were cognitively unable to meet the study requirements at the local investigator’s discretion. Once an eligible patient agreed to participate, the local investigator handed out a hard copy of the self-assessment questionnaire (BIOTICA), including a demographics section, and instructed the use of the electronic device. The local investigator advised patients to (i) complete the self-report questionnaire (BIOTICA) at home at the earliest convenience, ideally before taking the first dose, and (ii) to record every intake of the oral AB with the electronic device. The validated electronic device saved the date and time as soon as the patient confirmed the intake by pressing a button [32]. After treatment completion, participants posted the completed questionnaire with the electronic device back to the study centre. A member from the study team (MH or ES) contacted each patient for a follow-up telephone interview within two weeks of treatment completion. The telephone interview lasted five to ten minutes and assessed patient-reported health outcomes, pill count, strategies to implement the AB intake in daily life, side effects, and any issues with the electronic device. When the participants explained in a credible manner that they took the oral AB agent but forgot to activate the electronic device, for example, because they left the device at home, medication adherence data were enriched according to the obtained information. Participants were defined as lost to follow-up if three frustrating calls at different days and times were made, and no reaction occurred to a letter containing the written follow-up questions that was sent by post. The electronic data capture system RedCap^®^ was used to store and organise study data. It consisted of six parts; that is, eligibility questionnaire, demographics questionnaire, BIOTICA questionnaire, treatment details, follow-up interview, and end of study data.

### 2.5. Ethics Statement

This study was approved by the ethics committee of Northwestern Switzerland (EKNZ Project ID: 2020-00069) on 19 February 2020, and registered at clinicaltrial.gov (NCT04286230).

### 2.6. Statistical Analysis

Answers to the BIOTICA questionnaire were given on a five-point Likert scale (strongly agree—agree—neutral—disagree—strongly disagree), with points given values from zero to four. Reverse coding was performed for items where disagreement indicated a barrier. A higher score indicates a higher degree of perceived barriers. To investigate construct validity, which refers to the association between two instruments which should theoretically be related, the BIOTICA score was correlated with electronic monitored me-dication adherence data [33,34]. A total of 85 participants were aimed for to demonstrate a moderate effect correlation of r = 0.3 with a power of 80% and a type-one error alpha of 0.05. Significant, negative correlation coefficients were anticipated.

We used the following four medication adherence estimates to describe the implementation phase and applied the formula from Albert et al. [35]:

- taking-adherence [%]: (number of doses taken)/(number of doses prescribed) × 100;
- timing-adherence [%]: (number of doses taken within ±12.5% of the mean intake time)/(number of doses taken) × 100;
- consecutive taking adherence [%]: (maximum number of doses taken consecutively)/(number of doses prescribed) × 100;
- dose-to-dose interval [h min]: mean time difference between two consecutive doses of morning, noon, and/or evening intakes, where applicable.

Initiation of treatment was defined as taking the first dose on the day of medication dispensing by the pharmacy or of medication prescribing by the general practitioner (up to midnight). Discontinuation was defined as stopping the treatment at least two days before the prescribed end of treatment.

Microsoft Excel 2016 (Version: 16.0) [36] was used to calculate medication adherence estimates and RStudio was used for statistical analysis (Version: 4.0.2, year: 2020) [37]. Descriptive analysis was used for categorical values, which are presented as frequency (%), and for continuous variables, which are presented as mean and standard deviation (SD) or median and interquartile range (IQR), where applicable. Associations between medication adherence barriers, medication adherence estimates, and treatment characteristics were analysed with Spearman or Pearson correlations, Mann-Whitney-U-Test, or Kruskal-Wallis-Test, where appropriate. A correlation coefficient of r = 0.1 was interpreted as weak effect, r = 0.3 as moderate effect, and r = 0.5 as strong effect [38]. To assess reliability of the questionnaire, Cronbach’s alpha was calculated with acceptable values of 0.7–0.9 and a total item correlation with acceptable values > 0.2 [33,39]. Factor analysis with varimax rotation was conducted to identify the questionnaire’s underlying sub-scales and to reduce the number of items. The Kaiser-Meyer-Olkin measure of sampling and Barlett’s test of sphericity were applied to assess the eligibility of data for factor analysis. The number of factors was determined by eigenvalues > 1 and analysing the scree-plot. A cut-off value of 0.4 was used to exclude items from a particular subscale [40]. Electronically monitored adherence data were used to identify a suitable threshold of the BIOTICA questionnaire by conducting a receiver operating characteristic (ROC) analysis. A *p*-value of < 0.05 was considered statistically significant.

## 3. Results

### 3.1. Literature Search

The literature search was conducted on 10 February 2019, and generated 233 hits (Supplement 2). After removing duplicates, two researchers (AS, MH) screened titles, abstracts, and full-text articles. Twenty-two articles met the inclusion criteria and contri-buted to the qualitative synthesis of medication adherence barriers to ABs. Overall, 180 barriers were retrieved from the literature. Twenty-five barriers were categorised as unmodifiable, including age, gender, and level of education. From the remaining 155 modifiable barriers, the researchers removed synonyms (102) and conflated similar topics (20) so that 33 barriers were presented to the focus group discussion participants. Barriers belonged to 10 of the 11 domains of the TDF and included environmental context and resources (n = 13), knowledge (n = 4), social influence (n = 3), emotions (n = 3), beliefs about capabilities (n = 2), intentions (n = 2), memory (n = 2), skills (n = 2), beliefs about consequences (n = 1), and social, professional role and identity (n = 1). No barriers were categorised in the domain behavioural regulation (Supplement 3).

### 3.2. Focus Group Discussion

The FGD took place on March 27, 2019, with eight participants (mean age: 41 ± 20 years, women: 4), recruited from personal contacts, pharmacies, and the pharmacy department. AB treatment duration ranged from 5 to 10 days, and seven participants had to follow a twice-daily regimen. Participants mentioned 14 distinct barriers of which 3 diverged from the literature. These included (i) a feeling of personal failure when oral ABs are needed, (ii) difficulties taking the AB agent with meals, (iii) scared of allergies (Supplement 3). After prioritising all barriers, including the newly mentioned ones, seven barriers scored in the lowest quartile and were excluded from further processing according to their relevance. After conflating similar topics, a 16-item questionnaire was developed with single encompassing statements (Supplement 4).

### 3.3. Content Validity

Fourteen experts in the field of medication adherence (eight pharmacists, six physicians) participated in the survey that was disseminated on 31 October 2019. All items were acceptable regarding clarity (mean CVI: 0.94), ambiguity (mean CVI: 0.97), and comprehensibility (mean CVI: 1). The experts considered the item “saving some doses for the next time” irrelevant (I-CVI < 0.78), so that it was removed from further processing. Five items got an I-CVI < 0.78 and needed to be revised according to experts’ suggestions. Finally, a questionnaire with 15 items was created (Supplement 5, translated into English).

### 3.4. Construct Validity

Fourteen sites (11 pharmacies, 3 GP surgeries) participated in the construct validity study. All sites were located in Northwestern Switzerland (Basel-Land, Basel-Stadt, Aargau, and Zug). The recruitment period started on 21 February 2020, until 31 May 2021. The patient recruitment phase was interrupted for three months between March and June 2020 due to the covid-19 pandemic.

Overall, 82 patients were recruited, of which 14 were excluded from analysis due to missing adherence data or questionnaires. The analysis was performed with 68 complete datasets. The patient’s mean age was 51.53 (±16.7) years (range: 19–85 years), 60.3% were female (Table 1). Urinary tract infections (31.3%) and skin or soft tissue infections (21%) were the most frequent infections. The median treatment duration was 7 days (IQR: 5–10). The most frequently prescribed agents were amoxicillin/clavulanic acid (34%) and doxycycline (22%). A twice-daily regimen was prescribed in 60% of the cases (Table 1).

**Figure 1:**
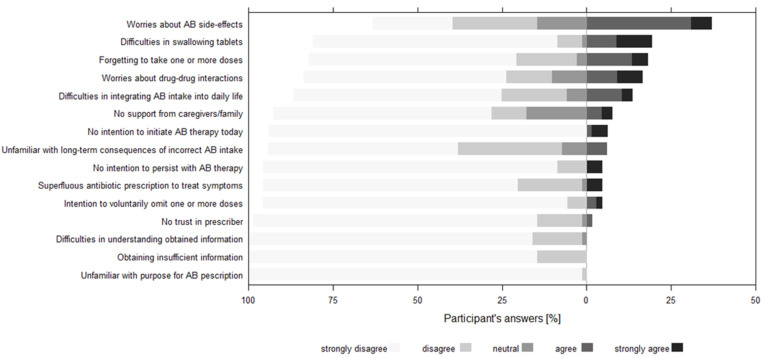
Frequencies of the answers to 15 medication adherence barriers to ABs, rated on a 5-point Likert-agreement scale (from strongly disagree to strongly agree) by 68 participants (67 for 4 items, respectively) sorted by descending order of agreement. The zero line represents the threshold between agreement and disagreement.

Overall, 42 (62%) patients reported between 1 and 7 barriers to medication adherence. The most frequently reported barriers were worries about side effects (37%), having dif-ficulties swallowing tablets (19%), and forgetting to take one or more doses (18%; Figure 1).

Overall, 79% of the patients initiated the AB treatment on the day of medicine dispensation and 19% the day after (Table 2). Overall, consecutive taking adherence was 88%, independent of the administration frequency (twice-daily regimen: 91%; thrice-daily regimen: 82%, *p* = 0.07). Six patients (8.8%) took more doses than prescribed, with a consecutive taking adherence between 103 and 133%. A consecutive taking adherence of 80–100% was reached by 63% of patients, 60–79% by 13%, 40–59% by 8.8%, and <39% by 5.9% of patients (data not shown).

The mean timing adherence was 85% (range: 35–100%) with significantly higher rates for a twice-daily (BID) regimen than a thrice-daily (TID) regimen (88% vs. 77%, *p* < 0.01). The dose-to-dose intervals were significantly shorter during the days than during the nights and independent of the frequency of administration (BID: 11 h 30 min vs. 12 h 30 min, *p* = 0.01; TID: 6 h 30 min vs. 7 h 2 min vs. 10 h 42 min, *p* < 0.001). All patients persisted with the treatment. The longer the treatment, the lower was the consecutive taking adherence (r = −0.24, *p* = 0.04). No other variables were associated with non-adherence (Supplement 6).

Five answers were missing on the returned questionnaires concerning item 5, 9, 11, 13, and 15, indicating that most items were generally clear and well understood by parti-cipants. For item 1 (“purpose of prescribed AB”), all but one patient reported, “strongly agree”, whereas for item 5 (“initiation”) and 10 (“voluntary omission of doses”), more than 90% of participants reported to “strongly agree”. This indicated a ceiling effect for item 1, 5, and 10.

The factor analysis was conducted on 15 items with orthogonal rotation (varimax). The Kaiser-Meyer-Olkin measure verified the sampling adequacy (overall KMO = 0.62), and Bartlett’s test of sphericity indicated that correlations between items were sufficiently large for factor analysis (chi^2^ = 294, *p* < 0.0001). According to the inflexions in the scree plot and the eigenvalues’ size, two components were retained for the final analysis. The eigenvalues of the two factors were 3.25 and 2.26, respectively. Factor 1 explained 23% of the variance and contained seven items. Based on the content of these seven items, the subscale represents rather practical barriers. The second factor contains five items, which account for 15% of the variance and represent rather perceptual barriers. Table 3 shows the factor loadings after rotation. Items 1, 4, and 14 had loadings below the cut-off value of 0.4 and were therefore excluded. An increase in Cronbach’s alpha supported the exclusion of item 4 (Table 4). Overall, 29% of participants had at least one barrier accounting for factor one (median score (IQR): 3 (0.75–5); range: 0–17) and 32% of participants had at least one barrier accounting for factor 2 (median score (IQR): 1 (0–3); range: 0–16; data not shown).

The score of the 15-item questionnaire (r =−0.25, *p* = 0.04; data not shown), as well as the score of the reduced 12-item questionnaire (r =−0.34, *p* < 0.01) and factor 1 (r = −0.38, *p* ≤ 0.001) correlated significantly with consecutive taking adherence rates (data not shown). All correlations coefficients were negative, indicating that a higher degree of adherence barriers measured with BIOTICA was associated with lower adherence levels, demonstrating construct validity. Cronbach’s alpha was 0.70 for the 12-item questionnaire and 0.69 for the 15-item version indicating acceptable internal reliability (Table 4).

The ROC analysis showed an Area Under the Curve (AUC) of 0.56 (95%CI 0.41–0.70) for the 15-item questionnaire and 0.57 (95% CI: 0.42–0.72) for the 12-item questionnaire. Based on the Youden-Index, the most suitable cut-off value for the 15-item questionnaire was 9 points (e.g., 3 to 4 barriers; sensitivity: 37%, specificity: 73%) and 7 points for the 12-item version (e.g., 2 to 3 barriers; sensitivity: 40%; specificity: 70%; data not shown). In our cohort, 23 participants (34%) exceeded a threshold of 9 points for the 15-item version and 25 participants (37%) exceeded a threshold of 7 points for the 12-item questionnaire.

## 4. Discussion

This study successfully developed a self-report 12-item questionnaire (BIOTICA) to assess medication adherence barriers to oral AB therapies. The questionnaire can be used to identify medication adherence barriers pre-emptively in an accurate and valid manner. Additionally, it gives hints about patients at risk for non-adherence at the point of medication dispense.

Since the WHO clearly reported that the inappropriate use of AB agents is one of the main causes of the growing bacterial resistance, antibiotic treatment is of utmost importance in an ambulatory setting. Healthcare providers in general and pharmacists, in particular, are encouraged to use their expertise to optimise medicines use and thus, improve patient health outcomes [24]. This is the primary aim of pharmaceutical care, that is, the pharmacist contribution to the full achievement of the benefits of medicines. In this context, adherence assumes particular importance, and community pharmacists need specific tools to tackle outpatients’ medicine intake behaviour. We generated a 12-item questionnaire named BIOTICA (BarrIers to Oral short-Term antibiotIC Adherence) using factor analysis with varimax rotation. Seven items clustering on factor 1 belong to practical barriers (such as social environment), and five items clustering on factor 2 represent perceptual barriers (such as worries) [41]. This corresponds to the broad dichotomous classification of adherence barriers described in the NICE adherence guidelines [42]. In addition, our practical barriers are consistent with the seven key themes identified through a systematic review that assessed medication adherence barriers [43].

Different estimates are available to describe the implementation phase of pharmacotherapy [35]. In an acute treatment setting, such as short-term use of antibiotics, we selected the consecutive taking adherence as the most accurate estimate because it indicates the maximal exposure to the medication linked to an uninterrupted intake behaviour. In our study, taking adherence tended to overestimate medication adherence when, for example, participants prolonged their treatment over the official treatment end and then reached taking adherence values superior to 100%. Taking adherence was also not sensitive enough to identify patients who missed doses in the middle of the treatment and added them after the official treatment end. Similarly, with the correctly dosed days, the first and last treatment days tended to be erroneous with a twice-daily or thrice-daily regimen, which are common dosing frequencies with antibiotic agents. The reason is that patients who initiated the treatment correctly, but late, on the day of dispense, might not be able to take all doses on that day, which is technically labelled as “incorrectly dosed day”.

Self-report adherence questionnaires tend to overestimate the intake behaviour [17,44]. However, questionnaires are adequate to assess beliefs and concerns [45] such as medication adherence barriers. Once healthcare providers have detected medication adherence barriers, they may help to overcome them, for example, with tailored interventions in patients at risk. A couple of interventions were studied to improve medication adherence to oral AB therapies in controlled trials. Interventions included the dispensing of ABs from the emergency ward instead of handing out a prescription [46], placing pharmaceutical pictograms on the package [47], or sending a mobile phone text message reminder to patients [48], among others, and did not influence medication adherence. On the contrary, dispensing the correct number of doses [49] and a pharmacist-led educational intervention with a leaflet [7] could significantly increase medication adherence. We assume that some interventions were unsuccessful because they did not target patients at risk for non-adherence and were not tailored to the patient’s various needs [50]. Using a short questionnaire such as BIOTICA might help identify key themes of risk and tailor interventions accordingly. Thus, a tailored intervention might be more likely to be successful.

Patient recruitment of this study started a couple of weeks before the start of the covid-19 pandemic and the initiation of governmental ordered lockdown regulations in Switzerland. The unique situation of a pandemic compromised our study in many ways. First, physicians and pharmacists were very engaged during the lockdown period with ensuring high-quality primary care and answering multiple questions of anxious patients. Study recruitment moved to the background to give way to urgent requirements. Second, patients were not willing to spend more time at the pharmacy/GP surgery than necessary in order to participate in a research study. Thus, recruiting was challenging.

To validate the BIOTICA questionnaire with a correlation of moderate effect size (correlation coefficient of 0.3), a sample size of 85 patients was set, considering that a conve-nient cohort of 60–70 patients has been used to validate adherence questionnaires [51,52,53], and taking into account a safety margin of 23% for high drop-out rates as reported in previous studies. Thus, 65 data sets would be sufficient for our purpose. Indeed, recruitment in primary care was extremely slow during the covid-19 pandemic and the dropout rate was high, with 10% of patients cancelling their participation once at home and 7% having technical issues. Nevertheless, we managed to recruit 82 patients in the given time, to obtain full datasets of 68 patients, and to calculate an overall effect size of r = 0.33. Our sample was also large enough to ensure a subject-to-item ratio of at least five for the factor analysis [54]. Moreover, four items had a loading > 0.6 for factor 1, which suggests that our sample size was sufficient for principal component analysis [55].

Our validation study is consistent with previous studies looking at adherence barriers in patients with HIV [28], atrial fibrillation [27], and with preventive cardiovascular medicine [29]. In our study, Cronbach’s alpha was 0.70 and close to the values from 0.72 to 0.82 obtained in these studies [27,28]. Furthermore, the correlation coefficients between BIOTICA and electronic monitoring was -0.34 in our study, which is comparable to correlations between −0.03 and −0.43 in similar studies [28,29].

The medication adherence of patients was surprisingly high in our study compared to other studies using electronic monitoring with adherence findings of 57% to 62% [17,44]. There are several explanations for this observation, such as the fact that patients changed their behaviour because of study participation (“Hawthorn effect”), as they obtained greater attention and care [56]. This was confirmed during the follow-up telephone interviews when some patients mentioned that “being monitored” helped them adhere to the treatment. Additionally, adherence to a short-term treatment against an acute infection might trigger a greater motivation to follow the treatment compared to a long-life treatment for a chronic illness. Further, the quantity and quality of information provided by pharmacists/GPs might have been more detailed than in usual care because of the study setting. Because healthcare providers had to explain the study’s purpose and procedure to the participants, including detailed information about the treatment, it is conceivable that they also emphasised medication adherence. Finally, as many patients were at home during the course of AB treatment (home-office, short-time work), this might have affected the medication intake behaviour and eventually increased medication adherence rates. In any case, we were able to correlate adherence barriers to a very strict measure of adherence.

Nevertheless, the question of how much adherence is enough for a favourable clinical outcome is still unanswered. When considering the pharmacological properties of the AB agents that are linked to the clinical success, ABs are categorised into time-dependent agents and concentration-dependent agents. For the first class, time over the minimal inhibitory concentration (MIC) is essential to reach the clinical outcome and multiple intakes are necessary over the whole day, especially if the half-life is short, such as for beta-lactams. Consequently, taking and timing adherence are equally important. For the second class, the peak serum concentration is crucial for clinical success. The dosing frequency is less critical. Thus, taking adherence might be more important than timing adherence, especially for medicine with a long half-life [57]. Therefore, the final interpretation of isolated missed doses must be performed in light of the pharmacokinetic properties of an antimicrobial agent [56]. Their impact on the clinical endpoint cannot be derived from mathematical formulas, as ingenious as they may be.

In Switzerland, antibiotic consumption in outpatients is low, with 9.1 defined daily doses (DDD) per 1.000 inhabitants per year, compared to the European average of 18.4 DDD per 1.000 inhabitants in 2019. The most frequently prescribed antibiotics in Switzerland are beta-lactamase inhibitors (40%), tetracycline (14%), and macrolides (14%) [58]. Thus, our study captured the most frequently prescribed antibiotics, with 34% of amoxicillin/clavulanic acid and 22% of doxycycline. We observed a marginal prescription of macrolides (3%). Our study participants suffered from urinary tract infections (31%) and skin infections (21%). The high consumption of doxycycline in our study might represent a seasonal effect. However, due to the covid-19 hygiene measures, few respiratory tract infections occurred during 2020, and therefore the number and classes of ABs consumed are likely to differ from other years.

Our 12-item questionnaire aligns with similar tools containing 10–18 items [28,29,59]. A 12-item questionnaire takes about five minutes to complete and is, therefore, convenient to use in daily practice [60]. We propose a five point Likert-scale as answer option, which is a compromise between the precision of the answers given by patients and the clinical need for a binary outcome, especially when an action is required according to the answer [33]. This dilemma can be approached by calculating a cut-off score with sensitivity and specificity values of the instrument, to define the threshold value above which an action is needed (BIOTICA: 40% sensitivity and 70% specificity). However, an alternative and pragmatic approach is to offer answer options as binary yes/no scale. This would allow clinicians to identify patients with barriers at first glance. However, the loss on precision of patient answers needs further investigation. Thus, our self-report questionnaire contributes to unveiling barriers to adherence before initiation of a short-term AB treatment and fills the literature gap. Implications for healthcare providers involved in prescribing and dispensing of oral ABs in primary care are manifold. For example, targeted interventions could be developed according to the answers to BIOTICA, in order to ameliorate the intake behaviour of oral ABs.

We acknowledge some strengths of this study. First, we selected community pharmacies and general practitioners who participated in former research projects. By doing this, we relied on experienced personal in patient recruitment and documenting. This process guarantees adequate patient inclusion and valid data. Second, we assessed medication adherence barriers immediately before initiated treatment. At this point, neither the physician nor the pharmacy staff could give additional information, so that barriers were not influenced. However, assessing medication adherence barriers before starting the treatment required some subjective projection about, for example, problems integrating the treatment into daily life or persistence. Although subjective, this reflection is crucial when healthcare professionals are to screen patients at risk for non-adherence at the point of medication dispense. Third, we used electronic monitoring to measure medication adherence. This is considered the gold standard in clinical trials because it delivers the most accurate results [61]. Fourth, we enriched medication adherence data with information from follow-up telephone interviews. By doing this, we ensured that missed doses were predominantly due to non-adherence to treatment (such as forgetfulness of intake) and not to the electronic device (such as having placed the device far from the medication). This approach allowed us to enrich medication adherence data with patient comments and, thus, calculate intake patterns as closely as possible to realities. Fifth, we selected the consecutive taking adherence to measure adherence, which is the most accurate estimate to describe the maximum exposure to the antimicrobial agents. By doing this, we narrowed the criteria for being non-adherent and enhanced the sensitivity of our analyses. Sixth, we formulated certain items inversely so that “disagreeing” indicates a barrier. Thus, we eliminated answering fatigue without affecting the comprehensibility of items.

We also acknowledge some limitations. First, we excluded 14 patients who did not return the electronic device (7), had technical problems (6), or did not complete the questionnaire (1), which reduced our sample size. Nevertheless, our validation study with 68 complete data sets remains robust. Second, we cannot exclude selection bias, which is inherent for observational studies. However, we diminished this bias by advising the pharmacists/GPs to ask every eligible patient to participate. Third, our results might not be generalisable to different patient groups, linguistic regions, or other countries. Our sample size was relatively small and restricted to outpatients living in Northwestern Switzerland. Fourth, we did not control for social desirability bias. However, patients were allowed to complete the questionnaire at home and not in the study setting premises, which might have helped to overcome this bias. In future validation studies, additional questions, such as the Social Desirability Scale-17 [62], could be added to the tested scale to control for social desirability. Fifth, we did not assess changes in perceived medication adherence barriers during the treatment [6]. Finally, we ignored what the prescribing physicians and the dispensing pharmacists told the patients about medication adherence barriers. It is conceivable that the information was more detailed than in usual care. Therefore, it is likely that even more medication adherence barriers are to be found in usual practice.

## 5. Conclusions

In conclusion, we successfully developed a self-report 12-item questionnaire to assess medication adherence barriers to short-term oral antibiotic treatment in outpatients. The questionnaire’s score correlated with medication adherence estimates and had a sensiti-vity of 70% in detecting patients at risk for non-adherence. Assessing medication adherence barriers in daily practice might help identify patients at risk for non-adherence, tailor interventions to the patient’s needs and concerns, and finally, contribute to the appropriate use of antimicrobial treatments. By doing this, healthcare providers such as community pharmacists or GPs might help ensure medication adherence in patients taking oral ABs and ultimately contribute to overcoming one of the six causes of antibiotic resistance. Future research may investigate further implications of BIOTICA in different medical services, for example a digitalised format for online services.

## Figures and Tables

**Table 1:** Demographic information and characteristics of the prescribed antibiotic treatment for patients of the construct validity study (*n* = 68).

**Patient Characteristics**	
Age (mean (SD) in years; range)	51.53 (±16.71); 19–85
Female (*n* (%))	41 (60.3)
Living alone (*n* (%))	16 (23.5)
Employed (*n* = 67) (*n* (%))	43 (63.2)
Level of education (*n* = 66) (*n* (%))	
primary	8 (11.8)
secondary	41 (60.3)
tertiary	17 (25.0)
Participants with comorbidities (*n* (%))	22 (32.3)
Co-medication (mean (SD); range)	1.94 (±2.64); 0–14
**Oral Antibiotic Treatment**	
Infection (*n* (%))	
Urinary tract infection	21 (31.3)
Skin or soft tissue infection	14 (21.0)
Lyme borreliosis	12 (17.9)
Not specified	8 (12.0)
Respiratory tract infection	7 (10.0)
Gastrointestinal infections	2 (3.0)
For prevention	2 (3.0)
others (ophthalmic (*n* = 1), venereal (*n* = 1)	2 (3.0)
Prescribed antibiotic (*n* (%))	
Amoxicillin/clavulanic acid	23 (33.8)
Doxycycline	15 (22.1)
Sulfamethoxazole-trimethoprim	6 (8.8)
Nitrofurantoin	5 (7.4)
Amoxicillin	4 (5.9)
Ciprofloxacin	3 (4.4)
Clindamycin	3 (4.4)
Norfloxacin	3 (4.4)
Cefuroxime	2 (2.9)
Clarithromycin	2 (2.9)
others (Clarithromycin/Amoxicillin, Cefpodoxime)	2 (2.9)
Dose regimen (*n* (%))	
BID	41 (60.3)
TID	25 (36.8)
QD	2 (2.9)
Duration of treatment (median (IQR) days; range)	7 (5–10); 3–30

Abbreviations: QD = once-daily, BID= twice-daily, TID = thrice-daily.

**Table 2:** Medication adherence estimates from the patients participating in the construct validation study (*n* = 68). Patients with a once-daily regimen (*n* = 2) were excluded from the sub-analysis. For estimates calculated, see statistical analysis in Section 2.

	Mean (SD)	Median (IQR)	Range	*p*-Value
**Taking adherence [%]**				
Overall (*n* = 68)	97.1 (±14.0)	100 (97.2–100)	33–133	
Twice-daily regimen (*n* = 38)	98.4 (±11.1)	100 (100–100)	50–133	0.132
Thrice-daily regimen (*n* = 21)	94.8 (±18.2)	100 (93.3–100)	33–133	
**Consecutive taking adherence [%]**				
Overall (*n* = 61)	87.8 (±24.6)	100 (73.3–100)	14–133	
Twice-daily regimen (*n* = 38)	90.9 (±22.4)	100 (100–100)	14–133	0.074
Thrice-daily regimen (*n* = 21)	81.8 (±28.0)	93 (67–100)	27–133	
**Timing adherence [%]**				
Overall (*n* = 61)	84.5 (±16.4)	89.6 (73.6–100)	35–100	
Twice-daily regimen (*n* = 38)	88.3 (±13.8)	92.3 (80–100)	43–100	0.005
Thrice-daily regimen (*n* = 21)	76.9 (±18)	78.6 (61–100)	35–100	
**Dose-to-dose interval BID [h:min]**				
morning-evening	11:30 (±55:16)	11:39 (11:04–11:57)	08:18–13:16	0.010
evening morning	12:30 (±54:14)	12:20 (12:01–12:56)	10:43–15:42	
**Dose-to-dose interval TID [h:min]**				
morning-noon	06:30 (±01:22)	06:50 (05:17–07:42)	04:02–08:21	<0.001
noon-evening	07:02 (±01:09)	07:06 (06:11–07:54)	04:25–10:01	
evening-morning	10:42 (±02:11)	10:51 (08:38–12:17)	07:59–15:15	
	**on the day of dispense**	**1 day after dispense**	**>1 day after dispense**	
Initiation (% (*n*))	79.4 (54)	19.1 (13)	1.47 (1)	
Persistence (% (*n*))	100 (68)			

SD = standard deviation; IQR = interquartile range.

**Table 3:** Summary of results from the exploratory factor analysis of the BIOTICA questionnaire (*n* = 68), with 15-items, translated from German. Item numbering refers to the order of the item in the questionnaire (Supplementary File, Supplement 5).

		Varimax Rotated Factor Loadings	
No.	Item	Factor 1	Factor 2
2	Obtaining insufficient information	0.86	
3	Difficulties in understanding obtained information	0.79	
13	No support from caregivers/family	0.65	
12	No trust in prescriber	0.63	
7	Unfamiliar with long-term consequences of incorrect AB intake	0.47	
11	Difficulties in integrating AB intake into daily life	0.46	
9	Forgetting to take one or more doses	0.44	
1	Unfamiliar with purpose for AB prescription	<0.4	
4	Superfluous antibiotic prescription to treat symptoms	<0.4	
8	No intention to persist with AB treatment		0.72
5	No intention to initiate AB treatment today		0.68
6	Difficulties in swallowing tablets		0.68
15	Worries about drug-drug interactions		0.59
10	Intention to voluntarily omit one or more doses		0.54
14	Worries about AB side-effects		<0.4
Eigenvalue		3.43	2.26
% of variance		23	15
Cronbach’s alpha		0.68	0.65

**Table 4:** Reliability assessment of the questionnaire (BIOTICA) using total item correlation coefficients and changes in Cronbach’s alpha if a particular item is deleted.

Item	15-Item Version (Cronbach’s Alpha: 0.69)		12-Item Version (Cronbach’s Alpha: 0.70)	
	Total Item Correlation Coefficient	Cronbach’s Alpha If Item Is Deleted	Total Item Correlation Coefficient	Cronbach’s Alpha If Item Is Deleted
**Item 1**: “Unfamiliar with purpose for AB prescription”	0.32	0.69	-	-
**Item 2**: “Obtaining insufficient information”	0.34	0.68	0.33	0.69
**Item 3**: “Difficulties in understanding obtained information”	0.25	0.68	0.26	0.69
**Item 4**: “Superfluous antibiotic prescription to treat symptoms.”	0.05	0.70	-	-
**Item 5**: “No intention to initiate AB treatment today”	0.27	0.67	0.30	0.68
**Item 6**: “Difficulties in swallowing tablets”	0.46	0.64	0.48	0.65
**Item 7**: “Unfamiliar with long-term consequences of incorrect AB intake”	0.26	0.68	0.26	0.69
**Item 8**: “No intention to persist with AB treatment”	0.36	0.67	0.37	0.67
**Item 9**: “Forgetting to take one or more doses”	0.44	0.65	0.42	0.66
**Item 10**: “Intention to voluntarily omit one or more doses”	0.39	0.66	0.40	0.67
**Item 11**: “Difficulties in integrating AB intake into daily life”	0.41	0.65	0.44	0.66
**Item 12**: “No trust in prescriber”	0.44	0.67	0.45	0.68
**Item 13**: “No support from caregivers/family”	0.29	0.67	0.31	0.68
**Item 14**: “Worries about AB side-effects”	0.26	0.68	-	-
**Item 15**: “Worries about drug-drug interactions”	0.28	0.68	0.23	0.70

## Data Availability

Data available on request to due privacy restrictions.

## Supplementary Materials

The following are available online at https://www.mdpi.com/article/10.3390/ijerph18157768/s1, Supplement 1: Search strategy; Supplement 2: Selection process of included studies; Supplement 3: Summary of barriers rated in the focus group discussion matched into the TDF; Supplement 4: Flow-chart of retrieving relevant medication adherence barriers to short therm oral antibiotic therapies in outpatients; Supplement 5: The self-report questionnaire (BIOTICA) [translated into English]; Supplement 6: Association between consecutive taking adherence and demographic variables.
