# Supplementary material for: Assessing Medication Adherence Barriers to Short-Term Oral Antibiotic Treatment in Primary Care—Development and Validation of a Self-Report Questionnaire (BIOTICA)
Source: Int J Environ Res Public Health. 2021 Jul 22;18(15):7768. doi: 10.3390/ijerph18157768 (PMC8345617; doi:10.3390/ijerph18157768)
Supplement: Supplementary file 1 [file ijerph-18-07768-s001.zip › ijerph-1271663-supplementary.pdf]

Supplementary Material

Supplement 1: Search strategy

Database	Date	Search terms
PubMed	03.05.19	1. patient compliance [MeSH and all fields]<br>2. non compliance (all fields)<br>3. noncompliance (all fields)<br>4. non-compliance (all fields)<br>5. medication adherence [MeSH and all fields)<br>6. non adherence (all fields)<br>7. nonadherence (all fields)<br>8. non-adherence (all fields)<br>9. 1 OR 2 OR 3 OR 4 OR 5 OR 6 OR 7 OR 8<br>10. Anti-Bacterial Agents [MeSH]<br>11. bacterial infections [MeSH]<br>12. antibiotic* (all fields)<br>13. antibiotic* therapy (all fields)<br>14. antibiotic* treatment (all fields)<br>15. antibiotic* regimen (all fields)<br>16. 10 OR 11 OR 12 OR 13 OR 14 OR 15<br>17. administration, oral [MeSH and all fields]<br>18. oral*<br>19. 17 OR 18<br>20. barrier* (all fields)<br>21. determinant* (all fields)<br>22. factor* (all fields)<br>23. 20 OR 21 OR 22<br>24. 9 AND 16 AND 19<br>25. Limit 14 to human
EMBASE	03.05.19	1. patient compliance (Emtree)<br>2. medication compliance (Emtree)<br>3. compliance (Emtree)<br>4. adherence (quick search)<br>5. medication adherence (quick search)<br>6. non compliance (quick search)<br>7. noncompliance (quick search)<br>8. non-compliance (quick search)<br>9. non adherence (quick search)<br>10. nonadherence (quick search)<br>11. non-adherence (quick search)<br>12. 1 OR 2 OR 3 OR 4 OR 5 OR 6 OR 7 OR 8 OR 9 OR 10 OR 11<br>13. antibiotic agent (Emtree)<br>14. bacterial infection (Emtree)<br>15. antibiotic therapy (Emtree)<br>16. antibiotic* treatment (quick search)<br>17. antibiotic* regimen (quick search)<br>18. 13 OR 14 OR 15 OR 16 OR 17<br>19. oral drug administration (Emtree)<br>20. barrier* (quick search)<br>21. determinant* (quick search)<br>22. factor* (quick search)<br>23. 20 OR 21 OR 22<br>24. 12 AND 18 AND 19 AND 23<br>25. limited to antibiotic agent and adolescent patients<br>26. Limit 12 to human

Cinhal	03.05.19	1. patient compliance (suggested subject term)<br>2. compliance (suggested subject term)<br>3. compliance to treatment (suggested subject term)<br>4. compliance to medication (suggested subject term)<br>5. adherence (suggested subject term)<br>6. adherence to medication (suggested subject term)<br>7. adherence to medication regiment (suggested subject term)<br>8. non compliance<br>9. noncompliance<br>10. non-compliance<br>11. non adherence<br>12. nonadherence<br>13. non-adherence<br>14. 1 OR 2 OR 3 OR 4 OR 5 OR 6 OR 7 OR 8 OR 9 OR 10 OR 11 OR 12 OR 13<br>15. antibiotics (suggested subject term)<br>16. antibiotic therapy (suggested subject term)<br>17. antibiotic treatment (suggested subject term)<br>18. antibiotic regimen<br>19. bacterial infection (suggested subject term)<br>20. 15 OR 16 OR 17 OR 18 OR 19<br>21. oral administration<br>22. oral<br>23. oral therapy<br>24. 21 OR 22 OR 23<br>25. barriers (suggested subject term)<br>26. obstacles (suggested subject term)<br>27. challenges (suggested subject term)<br>28. determinants (suggested subject term)<br>29. factors or causes of influences (suggested subject term)<br>30. 25 OR 26 OR 27 OR 28 OR 29<br>31. 14 AND 20 AND 24 AND 30<br>32. limited to humans, adults

Supplement 2: Selection process of included studies

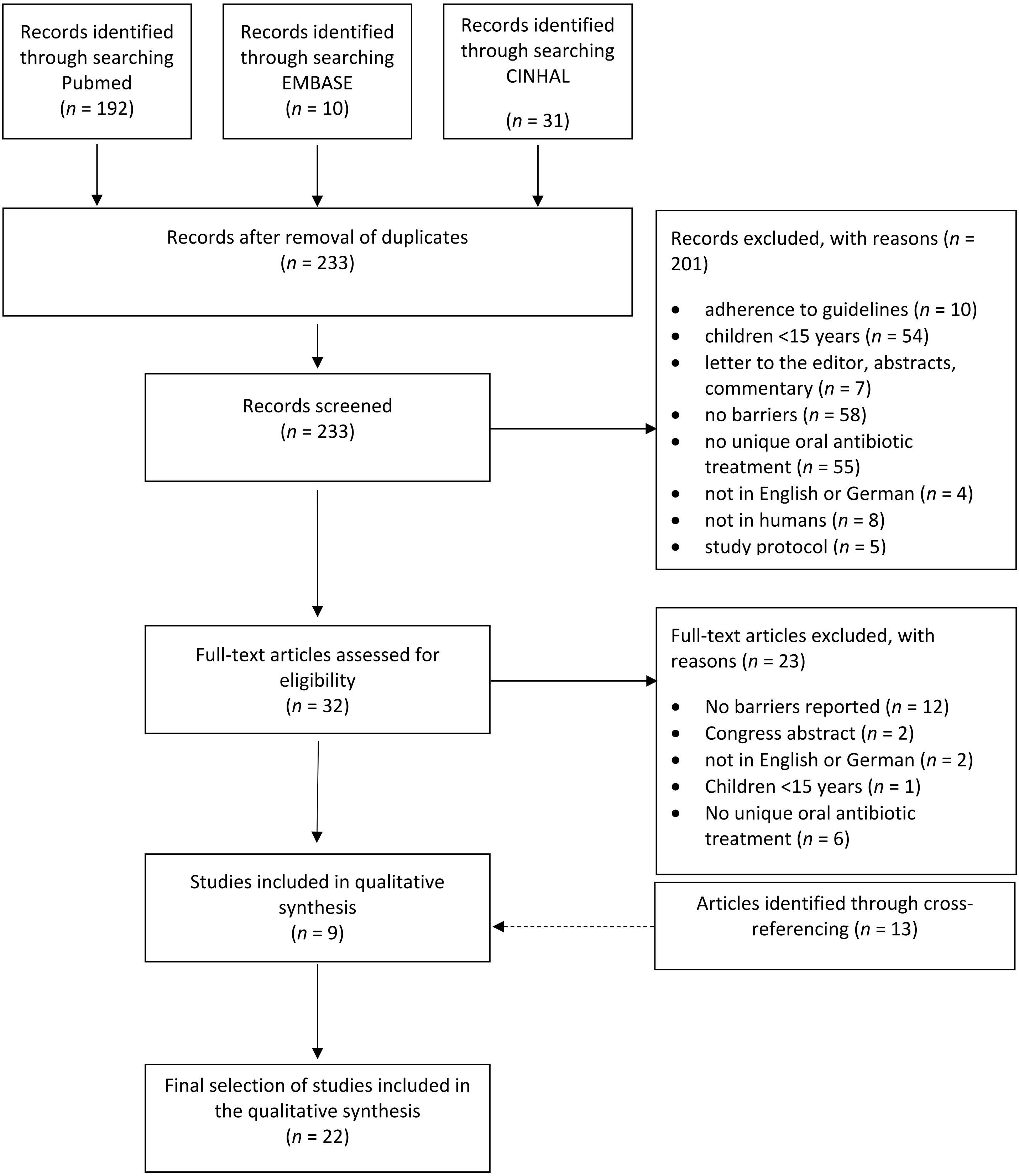

Supplement 3: Summary of barriers rated in the focus group discussion matched to the domains of the Theoretical Domains Framework (TDF) adapted by Allemann et al. [1] with references.

TDF domain	Identified barriers
1) Knowledge	• Not aware of the purpose of AB prescription [2], [FGD]<br>• Prescribed AB is superfluous to treat my symptoms [3, 4]<br>• Early discontinuation of treatment for any reason[2, 5-7]<br>• No knowledge about the consequences of not taking the oral AB as prescribed [8] [FGD]
2) Skills	• Swallowing difficulties [2, 6, 9] [FGD]<br>• Difficulties with opening the packaging [3, 10] [FGD]
3) Social/professional role and identity	• Trust in healthcare provider [2]
4) Beliefs about capabilities	• Perceived capability to fight the infection without antibiotics [2, 4]<br>• Save some doses for the next time [7] [FGD]
5) Beliefs about consequences	• Belief that medication is harmful (e.g. toxic, trouble) [2, 3, 10]
6) Intentions	• No intentions to start the treatment in a timely manner [3, 10-12]<br>• Voluntary omission of one or more doses [11]
7) Memory, attentionand decision process	• Forgetfulness [2, 4, 6, 7, 9-11, 13]<br>• Received information about the oral AB was difficult to understand [7]
8) Environmental context and resources	• Difficulties to integrate AB intake into daily life (4 sub-items: holiday, time change, irregular working hours, difficulties matching intakes with meals [new FGD]) [3, 9, 11, 13, 14] [FGD]<br>• Difficulties matching the AB intake with meals (2 sub-items: interval, duration) [6, 9, 14] [FGD]
Regimen	• pill burden (too many pills per day) [6]
Adverse events	• Occurrence of adverse effects (2 sub-items: Diarrhea, no appetite) [2-4, 6, 7, 12, 13, 15-20]
Integration and coordination of care	• Difficulty with accessing health care [2, 9, 13, 17] [FGD]
Financial aspects	• High costs of antibiotics [9, 12, 21, 22] [FGD]
9) Social influences	• Trust in healthcare provider [2]<br>• Support of caregivers and family [8] [FGD]<br>• Scared what others think of me taking oral AB [2]
10) Emotion	• A feeling of shame/embarrassment [23]<br>• Feeling of failure when having to take an antibiotic (new FGD)<br>• Scared of allergies (new FGD)<br>• Fear about side-effects [6, 7] [FGD]<br>• Fear about drug-drug interactions [3, 4, 11]
11) Behavioural regulation	-

References supplement 3

1. Allemann SS, Nieuwlaat R, van den Bemt BJ, Hersberger KE, Arnet I. Matching Adherence Interventions to Patient Determinants Using the Theoretical Domains Framework. *Front Pharmacol*. 2016;7:429.
2. Hirsch-Moverman Y, Shrestha-Kuwahara R, Bethel J, Blumberg H, Venkatappa T, Horsburgh C, et al. Latent tuberculous infection in the United States and Canada: who completes treatment and why? *The international journal of tuberculosis and lung disease : the official journal of the International Union against Tuberculosis and Lung Disease*. 2015;19(1):31-8.
3. Ho J, Taylor DM, Cabalag MS, Ugoni A, Yeoh M. Factors that impact on emergency department patient compliance with antibiotic regimens. *EMJ*. 2010;27(11):815-20.
4. West LM, Cordina M. Educational intervention to enhance adherence to short-term use of antibiotics. *Res Social Adm Pharm*. 2019;15(2):193-201.
5. Pechère J-C, Hughes D, Kardas P, Cornaglia G. Non-compliance with antibiotic therapy for acute community infections: a global survey. *Int J Antimicrob Agents*. 2007;29(3):245-53.
6. Lam F, Stevenson F, Britten N, Stell I. Adherence to antibiotics prescribed in an accident and emergency department: the influence of consultation factors. *Eur J Emerg Med*. 2001;8(3):181-8.
7. Yamamoto Y, Kadota J, Watanabe A, Yamanaka N, Tateda K, Mikamo H, et al. Compliance with oral antibiotic regimens and associated factors in Japan: compliance survey of multiple oral antibiotics (COSMOS). *Scandinavian journal of infectious diseases*. 2012;44(2):93-9.
8. McGrady ME, Brown GA, Pai AL. Medication adherence decision-making among adolescents and young adults with cancer. *European journal of oncology nursing : the official journal of European Oncology Nursing Society*. 2016;20:207-14.
9. Fernandes M, Leite A, Basto M, Nobre MA, Vieira N, Fernandes R, et al. Non-adherence to antibiotic therapy in patients visiting community pharmacies. *Int J Clin Pharm*. 2014;36(1):86-91.
10. Kardas P. Patient compliance with antibiotic treatment for respiratory tract infections. *Journal of Antimicrobial Chemotherapy*. 2002;49(6):897-903.
11. Faure H, Leguelinel-Blache G, Salomon L, Poujol H, Kinowski JM, Sotto A. Assessment of patient adherence to anti-infective treatment after returning home. *Médecine et Maladies Infectieuses*. 2014;44(9):417-22.
12. Kwara A, Herold JS, Machan JT, Carter EJ. Factors associated with failure to complete isoniazid treatment for latent tuberculosis infection in Rhode Island. *Chest*. 2008;133(4):862-8.
13. Eells SJ, Nguyen M, Jung J, Macias-Gil R, May L, Miller LG. Relationship between Adherence to Oral Antibiotics and Postdischarge Clinical Outcomes among Patients Hospitalized with staphylococcus aureus skin infections. *Antimicrobial agents and chemotherapy*. 2016;60(5):2941-8.
14. Cockburn J, Gibberd RW, Reid AL, Sanson-Fisher RW. Determinants of non-compliance with short term antibiotic regimens. *British medical journal (Clinical research ed)*. 1987;295(6602):814-8.
15. Katz BP, Zwickl BW, Caine VA, Jones RB. Compliance with antibiotic therapy for chlamydia trachomatis and neisseria gonorrhoeae. *Sex Transm Dis*. 1992;19(6):351-4.

16. Anastasio GD, Little JM, Jr., Robinson MD, Pettice YL, Leitch BB, Norton HJ. Impact of compliance and side effects on the clinical outcome of patients treated with oral erythromycin. *Pharmacotherapy*. 1994;14(2):229-34.
17. Machado Jr A, Finkmoore B, Emodi K, Takenami I, Barbosa T, Tavares M, et al. Risk factors for failure to complete a course of latent tuberculosis infection treatment in Salvador, Brazil. *The international journal of tuberculosis and lung disease : the official journal of the International Union against Tuberculosis and Lung Disease*. 2009;13(6):719-25.
18. Llor C, Bayona C, Hernandez S, Moragas A, Miravitlles M. Comparison of adherence between twice- and thrice-daily regimens of oral amoxicillin/clavulanic acid. *Respirology*. 2012;17(4):687-92. [eng]
19. Lee M, Kemp JA, Canning A, Egan C, Tataronis G, Farraye FA. A randomized controlled trial of an enhanced patient compliance program for Helicobacter pylori therapy. *Arch Intern Med*. 1999;159(19):2312-6.
20. Calvet X, Garcia N, Gene E, Campo R, Brullet E, Sanfeliu I. Modified seven-day, quadruple therapy as a first line Helicobacter pylori treatment. *Aliment Pharmacol Ther*. 2001;15(7):1061-5.
21. Ball AT, Xu Y, Sanchez RJ, Shelbaya A, Deminski MC, Nau DP. Nonadherence to oral linezolid after hospitalization: a retrospective claims analysis of the incidence and consequence of claim reversals. *Clin Ther*. 2010;32(13):2246-55.
22. Eastment MC, McClintock AH, McKinney CM, Narita M, Molnar A. Factors That Influence Treatment Completion for Latent Tuberculosis Infection. *J Am Board Fam Med*. 2017;30(4):520-7.
23. Thiboutot D, Gollnick H, Bettoli V, Dréno B, Kang S, Leyden JJ, et al. New insights into the management of acne: an update from the Global Alliance to Improve Outcomes in Acne group. 2009;60(5):S1-S50.

Supplement 4: Flow-chart of retrieving relevant medication adherence barriers to short therm oral antibiotic therapies in outpatients. Abbreviation: GD = Focus Group Discussion

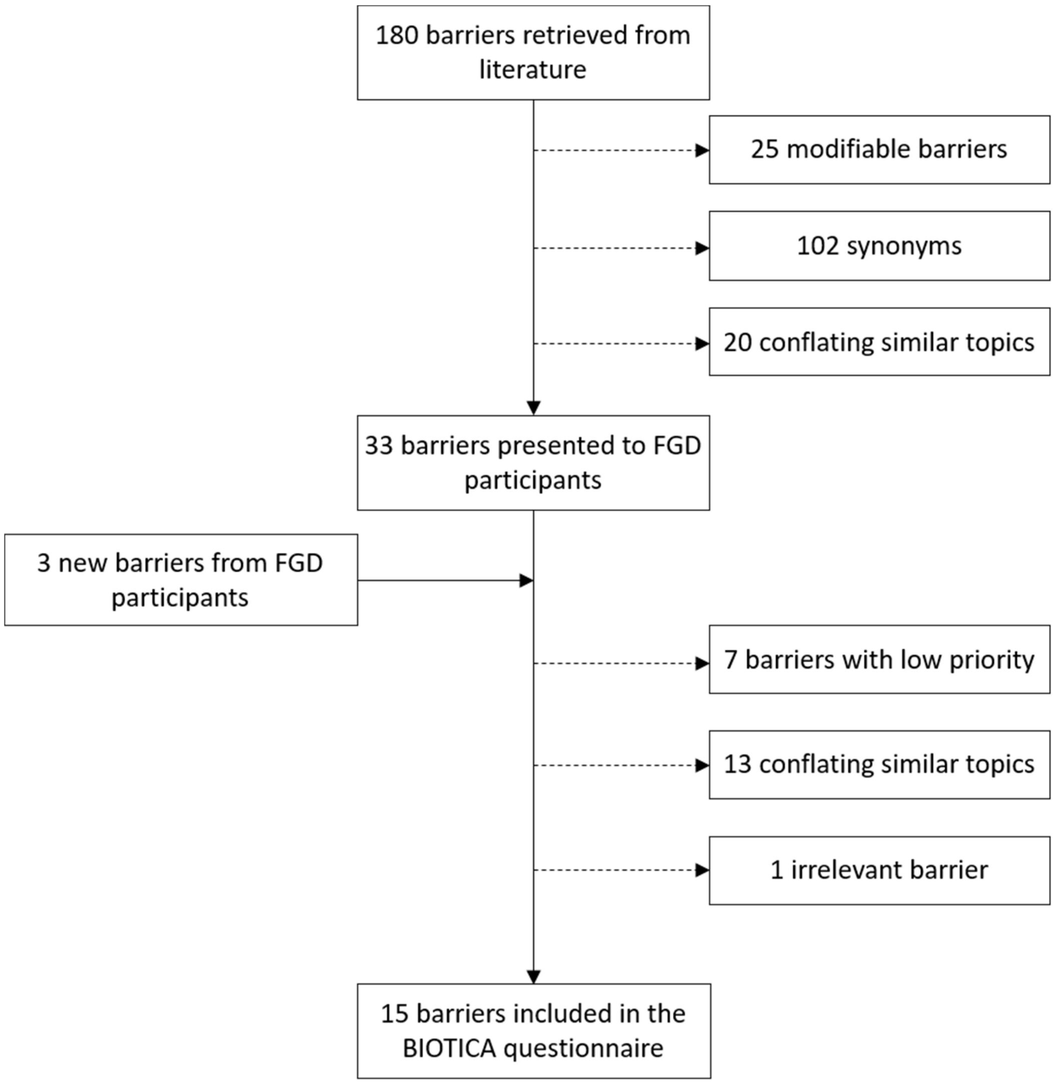

Supplement 5: The self-report questionnaire (BIOTICA) [translated into English]

Please rate to which extent the 15 statements apply to you by placing a cross after each statement for “strongly agree," "agree," "neutral," " disagree," or "strongly disagree." All questions refer to your current antibiotic therapy.

		**strongly agree**	**agree**	**neutral**	**disagree**	**strongly disagree**
1.	I know for what reason I have been prescribed an antibiotic.					
2.	I have received sufficient information to implement the therapy with the antibiotic correctly.					
3.	The information I received on the antibiotic was easy to understand.					
4.	I find the antibiotic superfluous for the treatment of my current ailments.					
5.	I will take the first dose of the antibiotic today.					
6.	I have difficulty swallowing tablets even with a glass of water.					
7.	I know the consequences for my health if I do not take the antibiotic correctly.					
8.	I will stop therapy earlier than prescribed.					
9.	I may forget to take one or more doses of the antibiotic.					
10.	I may deliberately skip taking one or more doses of the antibiotic.					
11.	I can easily incorporate the antibiotic treatment into my daily routine (e.g. combine it with activities).					
12.	I trust the prescribing doctor.					
13.	My environment (family, friends, caregivers) supports me during the antibiotic treatment.					
14.	I worry about side-effects					
15.	I worry about drug-drug interactions					

Thank you for answering the questions

Supplement 6: Association between consecutive taking adherence and demographic variables, with corresponding effect size (w: Mann-Whitney-U-Test, chi2: Kruskal-Wallis-Test, r: Spearman correlations).

	**Consecutive taking adherence [%]**		**effect size**	***p*-value**
**Variables**	**Mean (SD)**	**Median (IQR)**		
**co-medication**				
yes (*n* = 38)	87 (±25)	100 (32)	w: 435	0.74
no (*n* = 24)	91 (±23)	100 (0.07)		
**education**				
primary (*n* = 8)	98 (20)	100 (1.8)		
secondary (*n* = 32)	83 (28)	100 (37.8)	chi^2: 2.28	0.31
tertiary (*n* = 26)	93 (19)	100 (5.2)		
**symptoms**				
yes (*n* = 57)	88 (22)	100 (27)	w: 84.0	0.09
no (*n* = 5)	107 (15)	100 (0)		
**sex**				
female (*n* = 41)	90 (22)	100 (25)	w: 486	0.35
male (*n* = 27)	85 (29)	100 (33)		
**working**				
yes (*n* = 43)	87 (27)	100 (30)	w: 522	0.93
no (*n* = 24)	89 (19)	100 (13)		
**living alone**				
no (*n* = 52)	89 (25)	100 (13)	w: 382	0.59
yes (*n* = 16)	84 (25)	100 (30)		
**therapy duration (in days)**				
**age (in years)**			r: -0.24	*0.04*
			r: -0.01	0.92
